# The pore-forming subunit Kir6.1 of the K-ATP channel negatively regulates the NLRP3 inflammasome to control insulin resistance by interacting with NLRP3

**DOI:** 10.1038/s12276-019-0291-6

**Published:** 2019-08-06

**Authors:** Ren-Hong Du, Ming Lu, Cong Wang, Jian-Hua Ding, Guangyu Wu, Gang Hu

**Affiliations:** 1 Jiangsu Key Laboratory of Neurodegeneration, Department of Pharmacology, Nanjing Medical University, 101 Longmian Avenue, Nanjing, 211166 Jiangsu P. R. China; 2 Department of Pharmacology, Nanjing University of Chinese Medicine, 138 Xianlin Avenue, Nanjing, 210023 Jiangsu P. R. China; 3 Department of Pharmacology and Toxicology, Medical College of Georgia, Augusta University, 1459 Laney Walker Blvd., Augusta, 30912 Georgia USA

**Keywords:** Inflammasome, Insulin signalling, Diabetes, Experimental models of disease

## Abstract

Excessive activation of the NLRP3 inflammasome is a key component contributing to the pathogenesis of various inflammatory diseases. However, the molecular mechanisms underlying its activation and regulation remain poorly defined. The objective of this study was to explore the possible function of the K^+^ channel pore-forming subunit Kir6.1 in regulating NLRP3 inflammasome activation and insulin resistance. Here, we demonstrate that Kir6.1 depletion markedly activates the NLRP3 inflammasome, whereas enhanced Kir6.1 expression produces opposing effects both in mice in vivo and in primary cells in vitro. We also demonstrate that Kir6.1 controls insulin resistance by inhibiting NLRP3 inflammasome activation in mice. We further show that Kir6.1 physically associates with NLRP3 and thus inhibits the interactions between the NLRP3 inflammasome subunits. Our results reveal a previously unrecognized function of Kir6.1 as a negative regulator of the NLRP3 inflammasome and insulin resistance, which is mediated by virtue of its ability to inhibit NLRP3 inflammasome assembly. These data provide novel insights into the regulatory mechanism of NLRP3 inflammasome activation and suggest that Kir6.1 is a promising therapeutic target for inflammasome-mediated inflammatory diseases.

## Diabetes: Inflammation in insulin resistance

A potassium-regulating molecule, Kir6.1, blocks inflammation and prevents insulin resistance (IR), a hallmark of type 2 diabetes. Insulin triggers cellular glucose absorption, lowering blood glucose levels. In IR, cells resist insulin signals, causing unhealthy blood sugar levels. Inflammasomes, multi-protein complexes that assemble in response to infection and trigger an inflammatory response, were previously implicated in development of IR, but the details are poorly understood. Gang Hu at Nanjing Medical University in China and co-workers investigated how inflammation and IR are linked to Kir6.1. Mice lacking Kir6.1 showed high levels of inflammation and developed IR; treating the mice with Kir6.1 restored insulin sensitivity. Further study showed that Kir6.1 binds to one of the components of the inflammasome, preventing it from assembling. Kir6.1 shows promise for development of new treatments for inflammatory diseases, including IR.

## Introduction

Insulin resistance is a pathological complication and a hallmark of type 2 diabetes (T2D), characterized by an unresponsiveness of cells in the primary metabolic tissues (including liver, skeletal muscle, and adipose tissues) to the hormone insulin^1^. Inflammation accompanying excessive activation of inflammasomes, particularly the nucleotide-binding, oligomerization domain (NOD)-like receptor family, pyrin domain-containing 3 (NLRP3) inflammasome, represents a key component contributing to insulin resistance and has been implicated in the development of metabolic diseases, including T2D^2–6^.

Inflammasomes are large intracellular multiprotein complexes that play a crucial role in host defense, against a variety of pathogens^7,8^. Among several different inflammasome members identified thus far, the NLRP3 inflammasome has been intensively investigated. The NLRP3 inflammasome consists of three subunits, the scaffold NLRP3, the adaptor apoptotic speck protein, which contains a caspase recruitment domain (ASC) and the downstream effector procaspase-1, which can assemble in response to stimulation by pathogens and danger signals. A two-signal model has been proposed for the activation of the NLRP3 inflammasome, in which signal 1 or the priming signal is provided by microbial or endogenous molecules that activate the transcription factor NF-κB to induce the expression of NLRP3 and pro-interleukin (IL)-1β, whereas signal 2 or the NLRP3-specific activation signal activates the NLRP3 inflammasome to induce the cleavage of procaspase-1 into active caspase-1, which in turn cleaves the precursors pro-IL-1β and pro-IL-18 to produce mature, active IL-1β and IL-18, respectively^9,10^. Although a number of cellular events, such as Ca^2+^ signaling, ATP release, endoplasmic reticulum (ER) stress, mitochondrial reactive oxygen species, and lysosomal damage, have been suggested to activate the NLRP3 inflammasome, K^+^ efflux or a reduced intracellular K^+^ concentration is an unequivocal activation trigger^11–14^.

The intracellular K^+^ concentration is regulated by many proteins, including the Na^+^-K^+^ ATPases and K^+^ channels, which have direct, but opposing, effects. Among a number of K^+^ channels identified with distinct regulatory properties, ATP-sensitive K^+^ (K-ATP) channels belong to the inwardly rectifying class and are gated by the intracellular nucleotides ATP and ADP. K-ATP channels are hetero-octamers consisting of four pore-forming Kir6.x (Kir6.1 or Kir6.2) subunits and four regulatory sulfonylurea receptor (SUR1 or SUR2) subunits^15^. K-ATP channels couple metabolic energy to the cell membrane potential and regulate a variety of cellular responses, particularly those under metabolic stress, such as hyperglycemia, hypoglycemia, ischemia and hypoxia, and thus are considered a metabolic sensor. In particular, Kir6.2 and SUR1, which are highly expressed in the pancreas, constitute the K-ATP channel in pancreatic β-cells and control insulin secretion from β-cells in response to metabolically generated ATP. Naturally occurring gain- or loss-of-function mutations of Kir6.2 directly cause neonatal diabetes mellitus and congenital hyperinsulinism, respectively^16–18^. Kir6.1 is prominently expressed in vascular smooth muscle, skeletal muscle and glial cells, and its major function is to modulate the cellular response to insulin, rather than insulin release from β-cells^19–21^. In contrast to Kir6.2, the function of Kir6.1 in the development of insulin resistance and T2D remains unknown.

Therefore, the purpose of this study is to explore the role of the pore-forming subunit Kir6.1 of the K-ATP channels in the activation of the NLRP3 inflammasome and in insulin resistance. We reveal a previously unappreciated function of Kir6.1 as a negative regulator in the activation of the NLRP3 inflammasome and the development of insulin resistance in vivo and in vitro. Our studies also show that this function of Kir6.1 is likely mediated by its specific interaction with NLRP3, which in turn impairs the assembly of the NLRP3 inflammasome. These data provide novel insights into the regulatory mechanism of NLRP3 inflammasome activation and implicate Kir6.1 as a potential therapeutic target for inflammasome-mediated inflammatory diseases.

## Materials and methods

### Materials

ATP, monosodium urate (MSU), insulin, D-glucose, dexamethasone, type IV collagenase, the caspase-1 inhibitor AC-YVAD-CMK, lipopolysaccharide (LPS*, E. coli*, serotype 0111:B4), sodium palmitate (PA), fatty acid–free BSA, William’s E medium, and insulin-transferrin-sodium selenite supplement (ITS, 5 mg/l each) were purchased from Sigma (St. Louis, MO, USA). N-[1-(2,3-Dioleoyloxy)propyl]-N,N,N-trimethylammonium methyl-sulfate (DOTAP), complete protease inhibitor and X-tremeGENE HP DNA transfection reagent were obtained from Roche (Basel, Switzerland). dsDNA and flagellin were purchased from Invivogen (San Diego, CA, USA), and anthraxPA was obtained from Quadratech (Kattameya, Cairo, Egypt). Recombinant murine macrophage colony-stimulating factor (315-02) was purchased from PeproTech (Rocky Hill, NJ, USA). Anakinra was from Amgen (Thousand Oaks, CA, USA). Gentamicin and Lipofectamine 2000 were purchased from Invitrogen (Carlsbad, CA, USA). Antibodies against Kir6.1 (sc-11224), caspase-1 (sc-514), PYCARD (ASC, sc-271054), and protein A/G plus agarose were purchased from Santa Cruz Biotechnology (Santa Cruz, CA, USA), NLRP3 (AG-20B-0014-C100) from Adipogen (San Diego, CA, USA), phospho-AKT (4056) and AKT (4685) from Cell Signaling Technology (Danvers, MA, USA), and IL-1β (AF-401-NA) from R&D (Minneapolis, MN, USA). The plasmid pcDNA3-N-Flag-NLRP3 (75127) was obtained from Addgene (Watertown, MA, USA). A caspase-1 activity assay kit (ab39412) was obtained from Abcam (Cambridge, UK). All other materials were obtained as described elsewhere^22^.

### Mice

Kir6.1 knockout (KO) mice were generated as described previously^23^. Kir6.1 heterozygous mice were made available via the NIH-funded Mouse Mutant Regional Resource Center. These mice were subsequently mated to each other to generate homozygotes. KO of the gene encoding Kir6.1 was verified by PCR genotyping. Littermates generated from the same heterozygous breeding pairs were used as controls. All mice were backcrossed for ten generations to C57Bl/6J mice, and age-matched wild-type (WT) C57Bl/6J mice (12–16 weeks old) were used as controls in different experiments. Kir6.1 KO mice displayed normal body weight, behavior and activity levels, except for sudden death. The db/db mice and ob/ob mice on a C57Bl/6 background and their WT littermates were obtained from Jackson Laboratories. All mice were maintained under specific pathogen-free conditions and were treated in accordance with protocols approved by the Institutional Animal Care and Use Committee of Nanjing Medical University.

In the experiments measuring NLRP3 inflammasome activation, Kir6.1 KO mice were injected intraperitoneally (i.p.) with the caspase-1 inhibitor AC-YVAD-CMK at a dose of 40 mg/kg body weight daily for 7 days. In the experiments measuring insulin-mediated signaling, Kir6.1 mice were injected i.p. with insulin at a dose of 0.75 U/kg body weight. The liver and gastrocnemius muscle were collected and snap frozen in liquid nitrogen for analysis of AKT activation by immunoblotting.

### Generation and injection of lentiviruses expressing Kir6.1

Lentiviruses expressing mouse Kir6.1 in the lentiviral vector LV5 (LV5-mus-Kir6.1) or empty LV5 vector at a titration of 1 × 10^9 ^TU/ml were synthesized and packaged by Shanghai GenePharma Co., Ltd. (Shanghai, China). Kir6.1 KO and db/db mice at 12–16 weeks old (*n* = 6 in each group) were injected with 100 µl of lentivirus through the tail vein. Blood glucose was monitored daily in both groups. Mice were sacrificed at 7 days after injection, and the liver and the gastrocnemius muscle were prepared for immunoblot analysis. Blood samples were also collected, and serum glucose, insulin and IL-1β levels were measured by ELISA.

### Preparation, culture, transfection, and stimulation of bone marrow-derived macrophages (BMDMs)

BMDMs were isolated and cultured as previously described. Briefly, BMDMs were isolated from the tibia and femoral bone marrow of C57BL/6 mice and cultured with Dulbecco’s modified Eagle’s medium supplemented with 10% fetal bovine serum (FBS) and 20 ng/ml recombinant murine macrophage colony-stimulating factor. Culture fluid was exchanged with fresh culture medium every 3 days. Under these conditions, an adherent macrophage monolayer was obtained on day 7.

For transient transfection, BMDMs cultured to 70–80% confluence on 24-well plates were transfected with 1 µg of mouse Kir6.1 cDNA in the cDNA3.1 vector (pcDNA3.1-Kir6.1) or the pcDNA3.1 empty vector using X-tremeGENE HP DNA transfection reagent. After 6 h, the cells were cultured in complete growth media for 48 h.

To study the activation of the NLRP3 inflammasome, BMDMs were primed with 100 ng/ml LPS for 6 h and then stimulated with ATP (5 mM) for 30 min, MSU (250 µg/ml) for 6 h or palmitate-BSA (400 µM) for 24 h. To study the activation of the NLRP1, AIM2, and NLRC4 inflammasomes, BMDMs were treated with 500 ng/ml anthraxPA, 1 µg/ml dA/dT plus 2.5 µl/ml Lipofectamine 2000, or 20 µg/ml flagellin plus 25 µl/ml DOTAP, respectively.

### Isolation and treatment of primary hepatocytes

Primary hepatocytes were isolated by a two-step perfusion of type IV collagenase at a concentration of 0.2 mg/ml as described previously^24^. Hepatocytes were collected by centrifugation at 800 rpm for 8 min. Immediately after harvesting, the cells were suspended in prewarmed William’s E medium supplemented with 10% FBC, 20 ng/ml dexamethasone, ITS and 10 mg/ml gentamicin. The cells were plated in collagen-coated 25 cm^2^ flasks at a density of 1 × 10^6^ cells/well.

The cells were stimulated with macrophage conditioned medium in the absence or presence of the IL-1 receptor antagonist anakinra (1 µg/ml) for 24 h and then treated with insulin (200 nM) for 10 min. The cell lysates were prepared, and the activation of AKT was measured by immunoblotting using phospho-AKT antibodies.

### ELISA

IL-1β, TNF-α, IL-6, and IFN-γ levels in serum and in cell culture supernatants were measured by ELISA using a kit from R&D Systems (Minneapolis, MN, USA), whereas insulin in the fasting serum was measured by a kit from Millipore, according to the manufacturer’s instructions. The activation of caspase-1 in the liver was measured by a commercially available caspase-1 activity assay kit (ab39412, Abcam, Cambridge, UK) according to the manufacturer’s instructions.

### Analysis of intracellular K^+^ levels

BMDMs from WT and Kir6.1 KO mice were primed with 100 ng/ml LPS for 6 h and then stimulated with ATP (5 mM) for 30 min. The BMDMs were then extracted in ultrapure nitric acid before microwave digestion. The digested samples were diluted to 5% HNO_3_. Intracellular K^+^ was analyzed by a Perkin Elmer Optima 8000 inductively coupled plasma optical emission spectrometry (ICP-OES) spectrometer.

### Insulin tolerance test (ITT) and glucose tolerance test (GTT)

Mice were fasted for 4 h and then injected i.p. with insulin (0.75 U/kg body weight) and D-glucose (1.8 g/kg body weight). Glucose concentrations in tail blood were measured with a glucometer (Onetouch UltroEasy, Johnson & Johnson, Shanghai, China).

### Real-time quantitative RT-PCR (q-PCR)

Total RNA was extracted from the liver with TRIzol reagent (Invitrogen, Carlsbad, CA, USA). Reverse transcription PCR was carried out using a TAKARA PrimeScript RT reagent kit, and real-time PCR was performed using a QuantiTect SYBR Green PCR kit (Qiagen, Germany) with an ABI 7300 Fast Real-Time PCR System (Applied Biosystems, Foster City, CA, USA). GAPDH was used as an internal control for the real-time PCR amplification. The sequences of primers for real-time PCR analysis are as follows: GAPDH forward primer 5′-CAAAAGGGTCATCTCC-3′ and reverse primer 5′-CCCCAGCATCAAAGGTG-3′; Kir6.1 forward primer 5′- CGCAAACCCGAGTCTTCTAGGA-3′ and reverse primer 5′- CCTGGCCAACATCTTCCTTTCAC-3′.

### Coimmunoprecipitation (co-IP)

Co-IP was carried out as described previously^25,26^. Briefly, HEK293T cells cultured on 6-well microplates at a density of 2 × 10^5^ cells/well were cotransfected with 1 µg of N-terminally FLAG-tagged mouse NLRP3 in the pcDNA3 vector and mouse Kir6.1 in the pcDNA3.1 vector using Lipofectamine 2000 for 48 h. The cells were then lysed in lysis buffer containing 50 mM Tris-HCl at pH 7.4, 1% NP-40, 150 mM NaCl and Complete Protease Inhibitor (Roche, Basel, Switzerland). The cell lysates were incubated with anti-FLAG M2 magnetic beads overnight at 4 °C. For co-IP of endogenous proteins, the cell lysates were incubated with 2 µg of antibodies overnight at 4 °C and then incubated with protein A/G plus agarose beads for 2–4 h. After washing the beads with lysis buffer, the immunoprecipitated proteins were detected by immunoblotting.

### Immunoblotting

The tissues or cells were homogenized in lysis buffer (Beyotime, China), and the total lysates were separated by SDS-PAGE and transferred to a polyvinylidene difluoride membrane. Immunoreactive bands were detected by enhanced chemiluminescence plus detection reagent (Pierce, Rockford, IL, USA) and analyzed using an Omega 16ic Chemiluminescence Imaging System (Ultra-Lum, CA, USA).

### Statistical analysis

The differences between different treatments and genotypes were determined by one-way or two-way ANOVA, followed by Tukey’s post hoc test. Two independent samples were compared by Student’s *t* test, and differences were considered statistically significant at *p* < 0.05.

## Results

### Kir6.1 inhibits NLRP3 inflammasome activation in mice

**Fig. 1:**
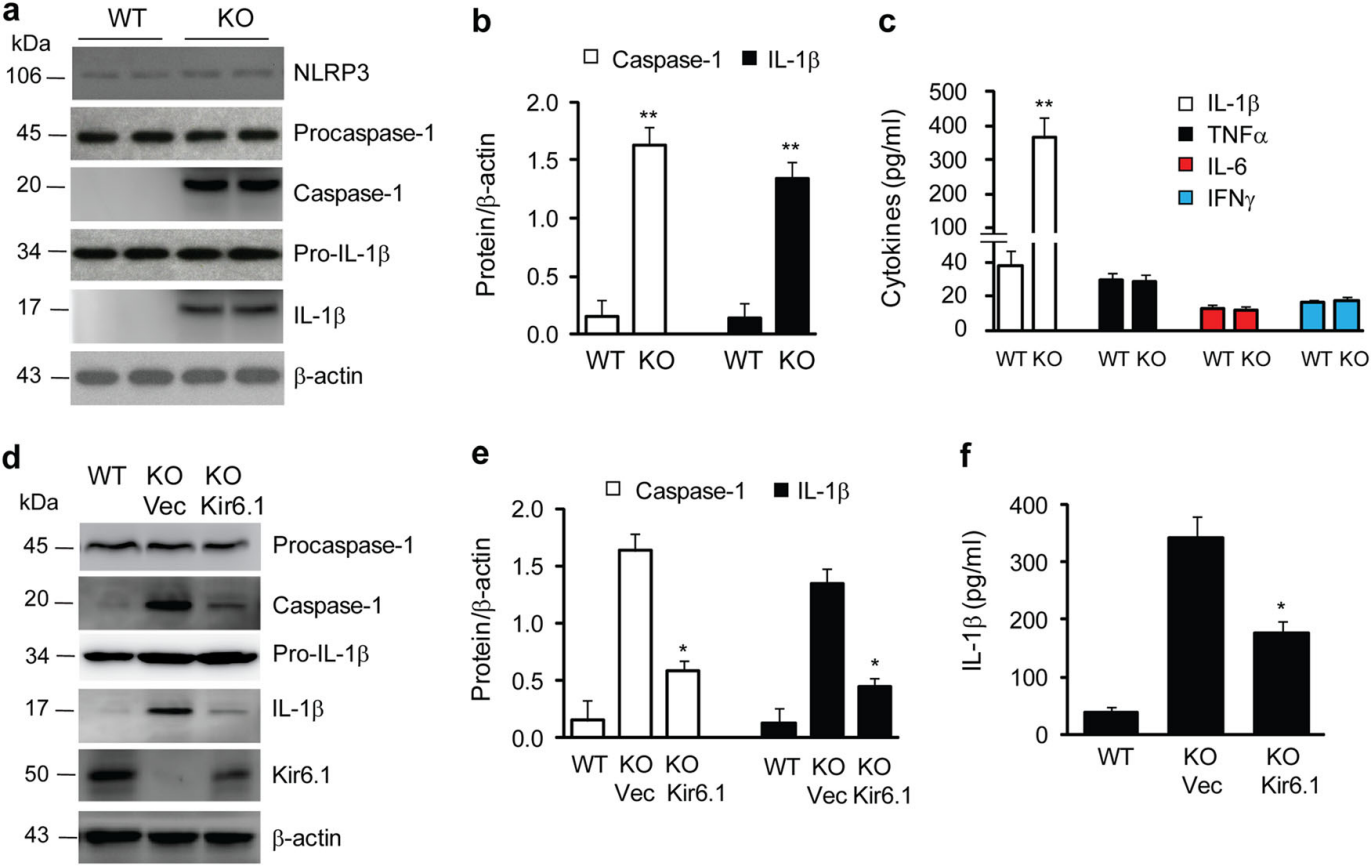
Kir6.1 deletion activates the NLRP3 inflammasome in the liver. **a** Representative immunoblots showing NLRP3 inflammasome activation in the liver of Kir6.1 KO mice. WT and KO are Kir6.1^+/+^ and Kir6.1^−/−^ mice, respectively. **b** Quantification of caspase-1 and IL-1β as shown in (**a**) (*n* = 6). **c** Serum IL-1β, TNF-α, IL-6, and INF-γ levels in WT and Kir6.1 KO mice as measured by ELISA (*n* = 12). The data shown are the mean ± SEM. **p < 0.01 vs WT. **d** Representative immunoblots showing the reduction in NLRP3 inflammasome activation in the liver of Kir6.1 KO mice by lentiviral expression of Kir6.1 for 7 days. **e** Quantification of data shown in (**d**) (*n* = 4). **f** Lentiviral expression of Kir6.1 reduced serum IL-1β (*n* = 6). The data shown are the mean ± SEM. *p < 0.05 vs KO mice injected with the parent vector (Vec)

The NLRP3 inflammasome is well known to activate caspase-1, which in turn induces the production of IL-1β. As an initial approach to directly investigate the role of Kir6.1 in the activation of the NLRP3 inflammasome, we used Kir6.1 KO mice to determine the effect of Kir6.1 depletion on the expression of caspase-1 and IL-1β. The levels of active caspase-1 and IL-1β (measured by immunoblotting) were markedly higher in livers from Kir6.1 KO mice than from control mice, whereas the levels of total NLRP3, procaspase-1 and pro-IL-1β were similar between the two genotypes (Fig. 1a, b).

We then determined if Kir6.1 deletion induced inflammation by measuring the production of proinflammatory cytokines in blood. Among several proinflammatory cytokines studied, including IL-1β, IL-6, tumor necrosis factor-α (TNF-α), and interferon-γ (IFN-γ), IL-1β was the only cytokine found to be present at significantly higher levels in the serum of Kir6.1 KO mice than in WT mice (as measured by ELISA) (Fig. 1c). These results clearly demonstrate that the deletion of Kir6.1 markedly activates the NLRP3 inflammasome and induces inflammation in mice in vivo.

We next determined whether the effect of Kir6.1 ablation on the NLRP3 inflammasome could be reversed by lentivirus-mediated expression of Kir6.1. Kir6.1 was clearly detected in the liver of Kir6.1 KO mice after intravenous injection of lentivirus expressing Kir6.1 for 7 days, albeit at a lower expression level than that of WT mice (Fig. 1d). Lentivirus-mediated Kir6.1 expression in Kir6.1 KO mice significantly reduced caspase-1 and IL-1β in the liver (Fig. 1d, e) and serum IL-1β (Fig. 1f). These data suggest that the enhanced activation of the NLRP3 inflammasome, as observed in Kir6.1 KO mice, is likely correlated with the expression level of Kir6.1.

### Kir6.1 inhibits NLRP3 inflammasome activation in cultured cells

**Fig. 2:**
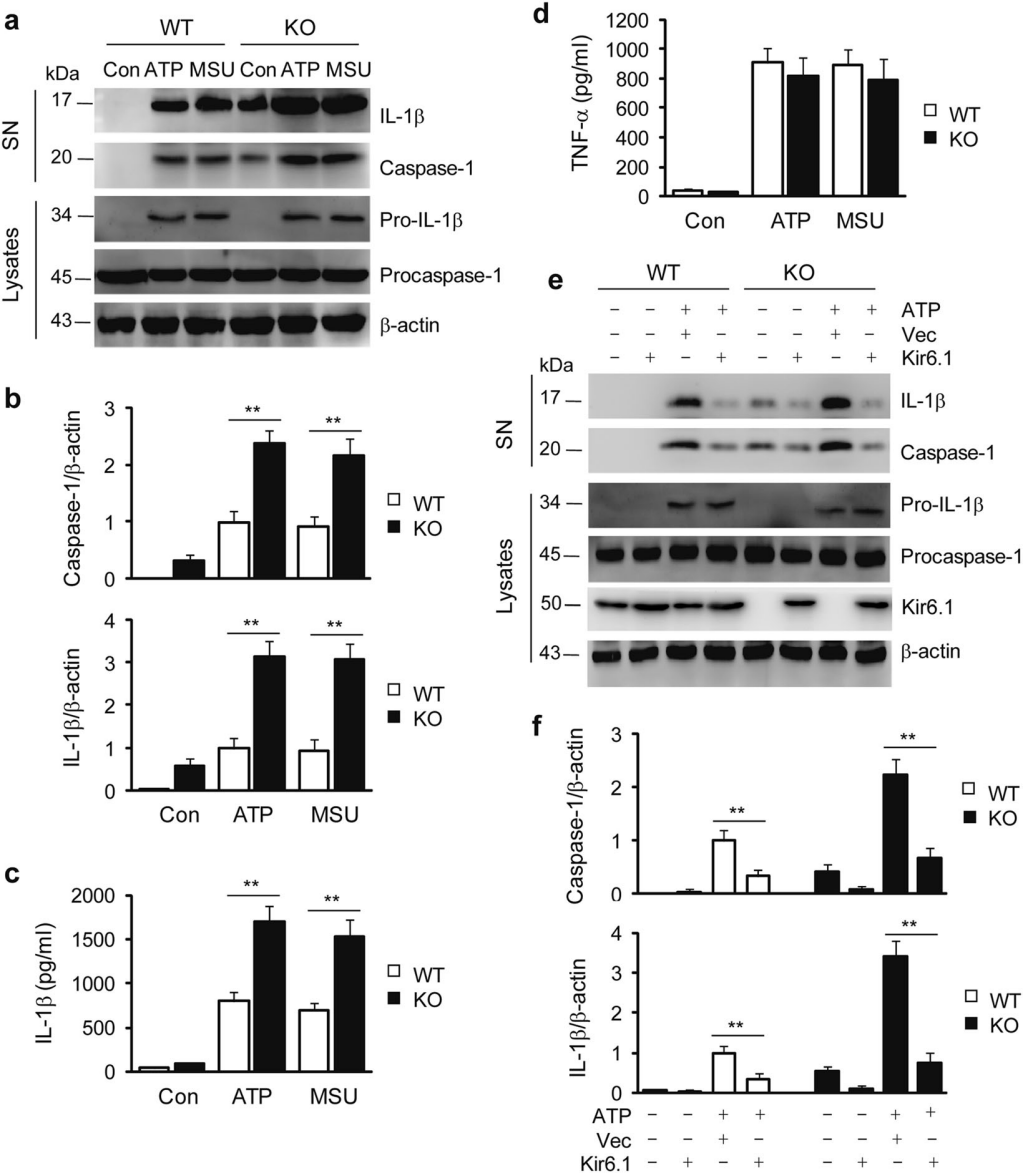
Kir6.1 ablation activates the NLRP3 inflammasome in BMDMs. **a** Representative immunoblots showing NLRP3 inflammasome activation in response to stimulation with LPS plus ATP and MSU in MBDMs isolated from WT and Kir6.1 KO mice. The levels of caspase-1 and IL-1β in the cell culture supernatants (SN) and procaspase-1 and pro-IL-1β in the total cell lysates were measured by immunoblotting. **b** Quantification of caspase-1 (upper panel) and IL-1β (lower panel), as shown in (**a**). **c**, **d** Concentrations of IL-1β (**c**) and TNF-α (**d**) in BMDMs were measured by ELISA. The data shown are the mean ± SEM from four independent experiments. **p < 0.01 vs WT. **e** Representative immunoblots showing the inhibition of NLRP3 inflammasome activation in BMDMs by transient expression of Kir6.1. BMDMs isolated from WT and Kir6.1 KO mice were transfected with Kir6.1 or control vector (Vec) for 48 h and then treated with LPS plus ATP. **f** Quantification of caspase-1 (upper panel) and IL-1β (lower panel), as shown in (**e**). The data shown are the mean ± SEM from four independent experiments. **p < 0.01 vs the cells transfected with control vector

Because macrophages are crucially involved in inflammation and thus secrete proinflammatory mediators, we used BMDMs to study the role of Kir6.1 in the activation of the NLRP3 inflammasome in vitro. Consistent with our data obtained in the livers of Kir6.1 KO mice, the activation of caspase-1 and the secretion of IL-1β, either at basal levels or in response to stimulation with LPS plus ATP and MSU or PA-BSA, were markedly higher in BMDMs from Kir6.1 KO mice than BMDMs from WT mice, as measured by immunoblotting (Fig. 2a, b, Fig. s1). The production of IL-1β (Fig. 2c), but not TNF-α (Fig. 2d), was also increased in BMDMs from Kir6.1 KO mice as measured by ELISA. In marked contrast, the activation of caspase-1 and the secretion of IL-1β were indistinguishable in BMDMs from Kir6.1 KO and WT mice after activation of the NLRP1, NLRC4, and AIM2 inflammasomes (Fig. s2). Furthermore, the activation of caspase-1 and the secretion of IL-1β at the basal level and in response to ATP stimulation in BMDMs from both Kir6.1 and WT mice was almost completely blocked by transient expression of Kir6.1 (Fig. 2e, f). These data suggest that Kir6.1 ablation potently enhances the activation of the NLRP3 inflammasome not only in mice in vivo but also in cultured cells in vitro, and the actions of Kir6.1 on the NLRP3 inflammasome are likely specific.

### Channel-independent actions of Kir6.1 on NLRP3 inflammasome activation

**Fig. 3:**
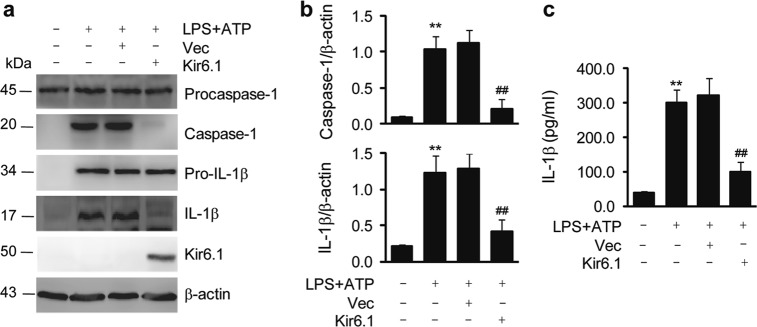
Kir6.1 inhibits NLRP3 inflammasome activation in A549 cells. **a** Inhibition of the NLRP3 inflammasome by Kir6.1 in A549 cells. A549 cells were transfected with Kir6.1 or vector alone for 48 h and then treated with LPS plus ATP. **b** Quantification of caspase-1 (upper panel) and IL-1β (lower panel), as shown in (**a**). **c** Concentrations of IL-1β in A549 cells measured by ELISA. The data shown are the mean ± SEM from four independent experiments. **p < 0.01 vs cells with no stimulation and transfection and ^##^*p* < 0.01 vs cells stimulated with LPS + ATP and transfected with vector

Our data obtained from cultured BMDMs suggested that the action of Kir6.1 on the activation of the NLRP3 inflammasome depends on its mere presence and perhaps does not require K-ATP channel function. To substantiate this possibility, we sought to determine the effect of Kir6.1 on the activation of the NLRP3 inflammasome in a cellular environment in which Kir6.1 is unable to form K-ATP channels. For this purpose, we chose A549 cells since previous studies have shown that they do not express endogenous K-ATP channels^27^. Consistently, the K-ATP channel subunits SUR1, SUR2A, SUR2B, Kir6.1, and Kir6.2 were undetectable by immunoblotting in A549 cells in our system (Fig. s3). We then determined the effect of transient expression of Kir6.1 on the activation of caspase-1 and the secretion of IL-1β in A549 cells. Stimulation with LPS plus ATP markedly enhanced the activation of caspase-1 and the secretion of IL-1β as measured by immunoblotting and ELISA (Fig. 3a–c), indicative of NLRP3 inflammasome activation. Transient expression of exogenous Kir6.1, but not the control vector, almost completely blocked caspase-1 activation and IL-1β production in response to stimulation with LPS and ATP in A549 cells (Fig. 3a–c). In addition, the intracellular K^+^ level, in response to stimulation with LPS plus ATP, was indistinguishable in BMDMs from Kir6.1 KO and WT mice (Fig. s4). These results demonstrate that enhanced expression of Kir6.1, without other K-ATP channel subunits, is sufficient to inhibit the NLRP3 inflammasome and suggest that the action of Kir6.1 on NLRP3 inflammasome activation is independent of its K-ATP channel function.

### Kir6.1 ablation results in insulin resistance in mice

**Fig. 4:**
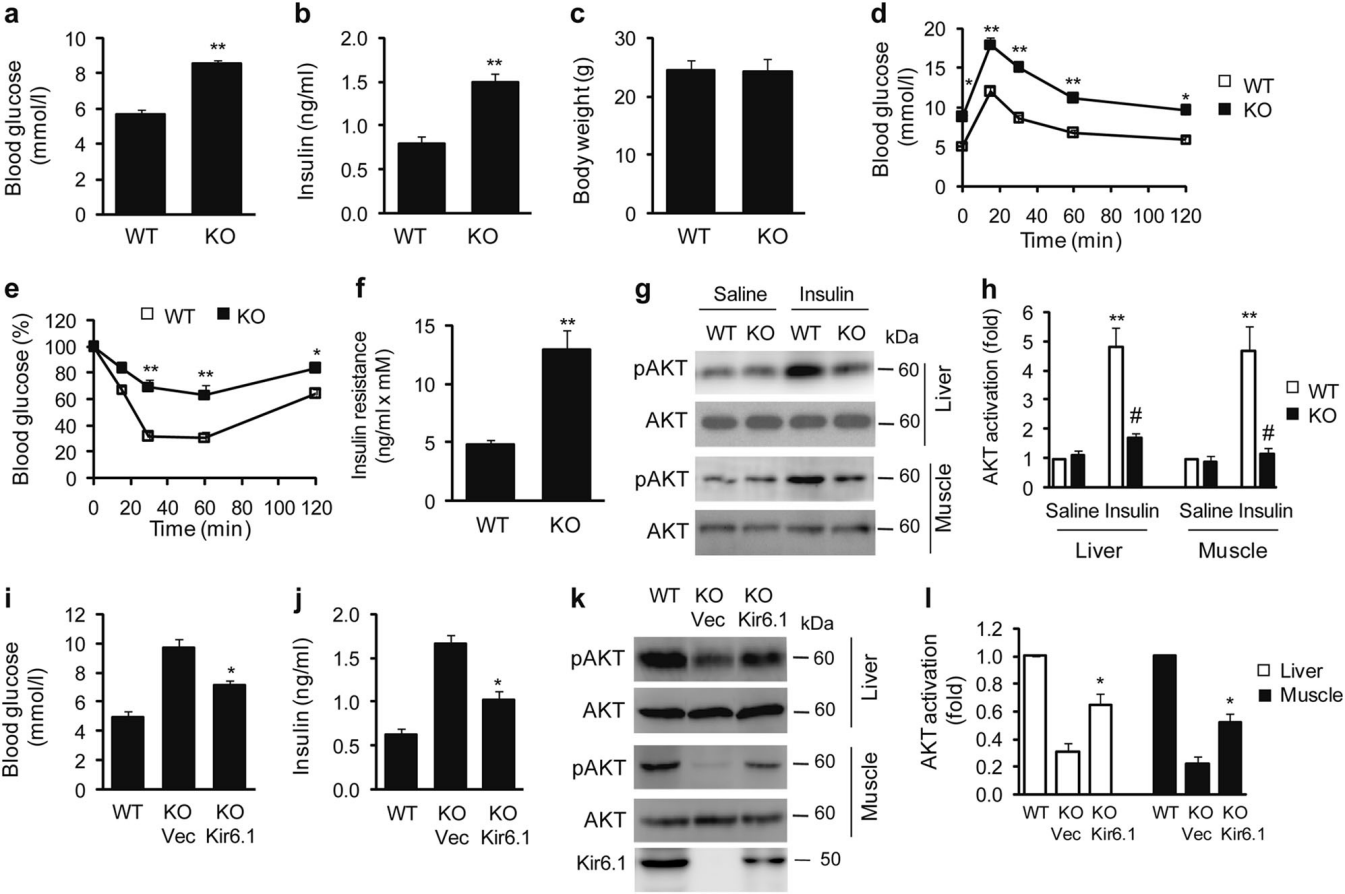
Kir6.1 ablation leads to insulin resistance. **a**–**c** Basal blood glucose (**a**) and fasting serum insulin levels (**b**) and body weight (**c**) in WT and Kir6.1 KO mice after 4 h of fasting (*n* = 12). **d** Oral glucose tolerance tests. Mice were fasted for 4 h prior to intraperitoneal injection of D-glucose (1.8 g/kg body weight) (*n* = 10). **e** Insulin tolerance tests. Mice were fasted for 4 h prior to intraperitoneal injection of insulin (0.75 U/kg body weight) (*n* = 10). **f** HOMA-IR index of insulin sensitivity (HOMA = insulin [ng/ml] × glucose [mM]) (*n* = 12). The data are presented as the mean ± SEM. *p < 0.05 and **p < 0.01 vs WT. **g** Representative immunoblots showing inhibition of insulin-induced AKT activation by Kir6.1 knockout in the liver and skeletal muscle. The mice were injected with either saline or insulin (0.75 mU/g body weight) for 10 min. **h** Quantification of data shown in (**g**); *n* = 4. The data are presented as the mean ± SEM. **p < 0.01 vs. the corresponding saline group; ^#^*p* < 0.05 vs. WT mice injected with insulin. **i**, **j** Lentiviral expression of Kir6.1 reduced blood glucose (**i**) and insulin levels (**j**) in Kir6.1 KO mice; *n* = 6. **k** Kir6.1 expression enhanced AKT activation in the liver and muscle of Kir6.1 KO mice. **l** Quantification of data shown in (**k**); *n* = 4. The data are presented as the mean ± SEM. *p < 0.05 vs Kir6.1 KO mice injected with vector

Because the activation of the NLRP3 inflammasome and the production of proinflammatory cytokines is closely associated with insulin resistance, we next studied whether Kir6.1 KO induced insulin resistance in mice. Blood glucose (Fig. 4a) and insulin levels (Fig. 4b) were significantly higher in Kir6.1 KO mice than their WT counterparts, whereas the body weights were similar (Fig. 4c). Blood glucose levels measured in glucose and ITTs (Fig. 4d, e) were also considerably higher over time in Kir6.1 KO mice, indicative of the impairment of glucose metabolism and insulin sensitivity. The quantification of insulin resistance based on the homeostasis model assessment (HOMA: insulin [ng/ml] × glucose [mM]) showed that insulin resistance was increased by approximately twofold in Kir6.1-null mice compared to WT mice (Fig. 4f).

We subsequently determined whether Kir6.1 KO could affect insulin-mediated signaling by measuring the activation of AKT, one of the most important kinases downstream of insulin and the insulin receptor. As expected, insulin administration markedly enhanced the activation of AKT in both liver and skeletal muscle tissues of WT mice (Fig. 4g, h). In contrast, insulin-stimulated AKT activation was dramatically reduced in Kir6.1 KO mice, compared with WT mice. These data clearly demonstrate that the deletion of the Kir6.1 gene induces insulin resistance in mice.

We next determined whether the effect of Kir6.1 ablation on insulin sensitivity could be reversed by lentivirus-mediated overexpression of Kir6.1. Blood glucose (Fig. 4i) and insulin levels (Fig. 4j) were significantly lower in Kir6.1 KO mice administered Kir6.1 lentivirus than KO mice treated with control virus. Similarly, insulin-mediated activation of AKT was significantly augmented by lentiviral expression of Kir6.1 in the liver and skeletal muscle (Fig. 4k, l). These data suggest that, similar to the activation of the NLRP3 inflammasome, insulin resistance observed in Kir6.1 KO mice can be reversed by the expression of Kir6.1.

### Inhibition of the NLRP3 inflammasome improves insulin resistance in Kir6.1 KO mice

**Fig. 5:**
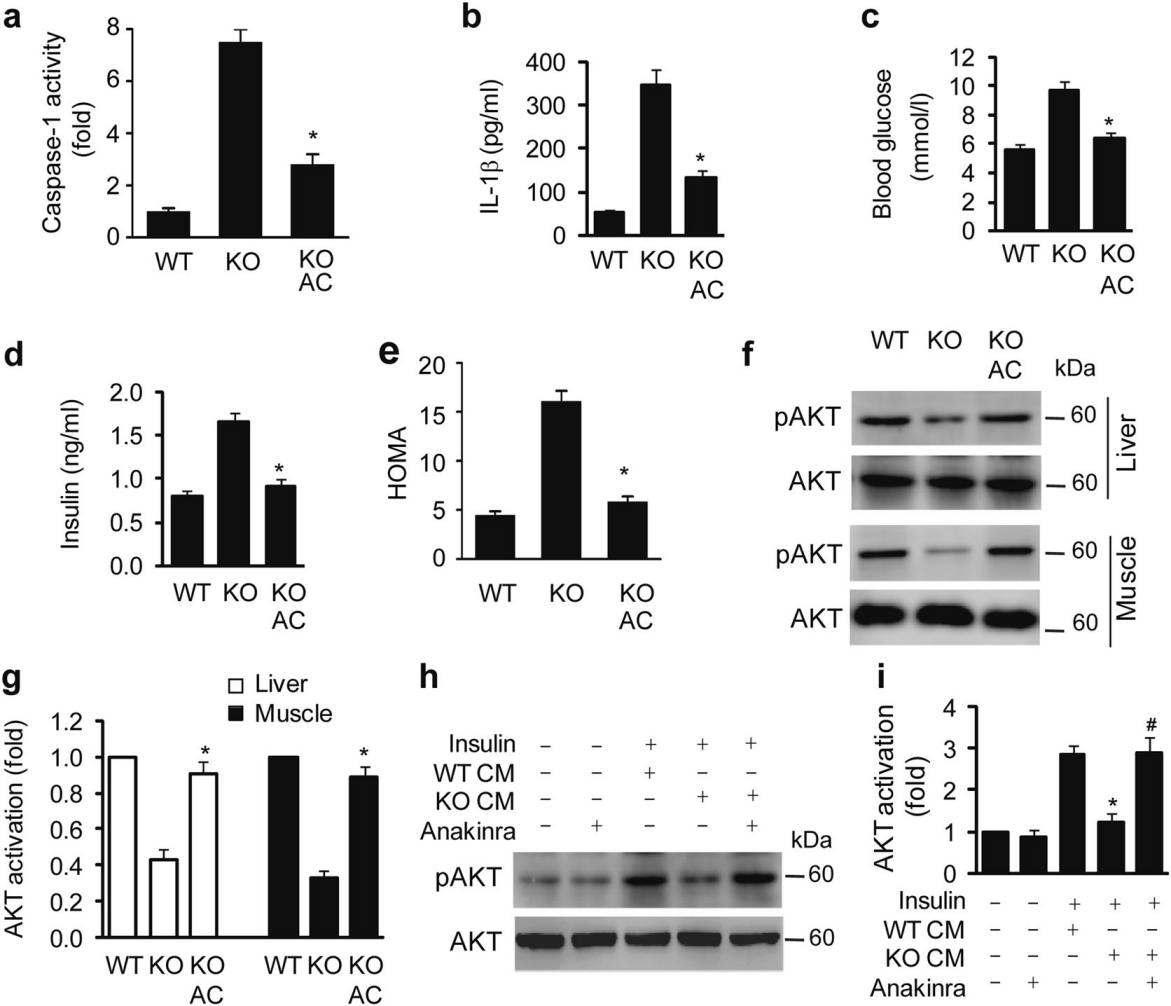
Pharmacological inhibition of the NLRP3 inflammasome prevents insulin resistance in Kir6.1 KO mice. **a–e** The caspase-1 inhibitor AC-YVAD-CMK (AC) inhibited caspase-1 activity (**a**), serum IL-1β (**b**), basal blood glucose (**c**), fasting serum insulin (**d**), and HOMA (**e**) in Kir6.1 KO mice; *n* = 6. **f** AC-YVAD-CMK enhanced AKT activation in the liver and muscle of Kir6.1 mice. **g** Quantification of data shown in (**f**); *n* = 4. Kir6.1 KO mice were intraperitoneally injected with AC-YVAD-CMK at a concentration of 40 mg/kg body weight for 7 days. The data are presented as the mean ± SEM. *p < 0.05 vs KO. **h** Representative immunoblots showing the effect of anakinra on insulin-mediated AKT activation. Primary hepatocytes were pretreated with conditioned medium (CM) generated from WT and Kir6.1 KO macrophages for 24 h in the absence or presence of anakinra and then stimulated with insulin (200 nM) for 10 min. **i** Quantification of shown in (**h**). The data shown are the mean ± SEM from four independent experiments. *p < 0.05 vs. cells treated with WT CM; ^#^*p* < 0.05 vs. cells treated with KO CM

To further determine whether insulin resistance observed in Kir6.1 KO mice was indeed caused by enhanced NLRP3 inflammasome activation, Kir6.1 KO mice were treated with the caspase-1-specific inhibitor AC-YVAD-CMK via intraperitoneal injection at a dose of 40 mg/kg for 7 days, and blood glucose and insulin levels were subsequently measured. AC-YVAD-CMK treatment markedly decreased caspase-1 activity (Fig. 5a), serum IL-1β (Fig. 5b), blood glucose (Fig. 5c), fasting serum insulin levels (Fig. 5d), and HOMA, an indicator of insulin resistance (Fig. 5e). Moreover, this treatment significantly enhanced AKT activation in the liver and skeletal muscle (Fig. 5f, g).

By using primary cultures of hepatocytes as a cell model, we next determined whether pharmacological inhibition of IL-1β could enhance insulin sensitivity in vitro. Insulin-mediated activation of AKT was markedly inhibited by conditioned medium generated from macrophages isolated from Kir6.1 KO mice, and this inhibition was completely reversed by treatment with the IL-1 receptor antagonist anakinra (Fig. 5h, i). These data suggest that pharmacological inhibitors of the NLRP3 inflammasome signaling pathway are able to attenuate the insulin resistance induced by Kir6.1 ablation in mice in vivo and in cultured cells in vitro.

### Kir6.1 is downregulated in db/db mice, and increased Kir6.1 expression ameliorates insulin resistance in these animals

**Fig. 6:**
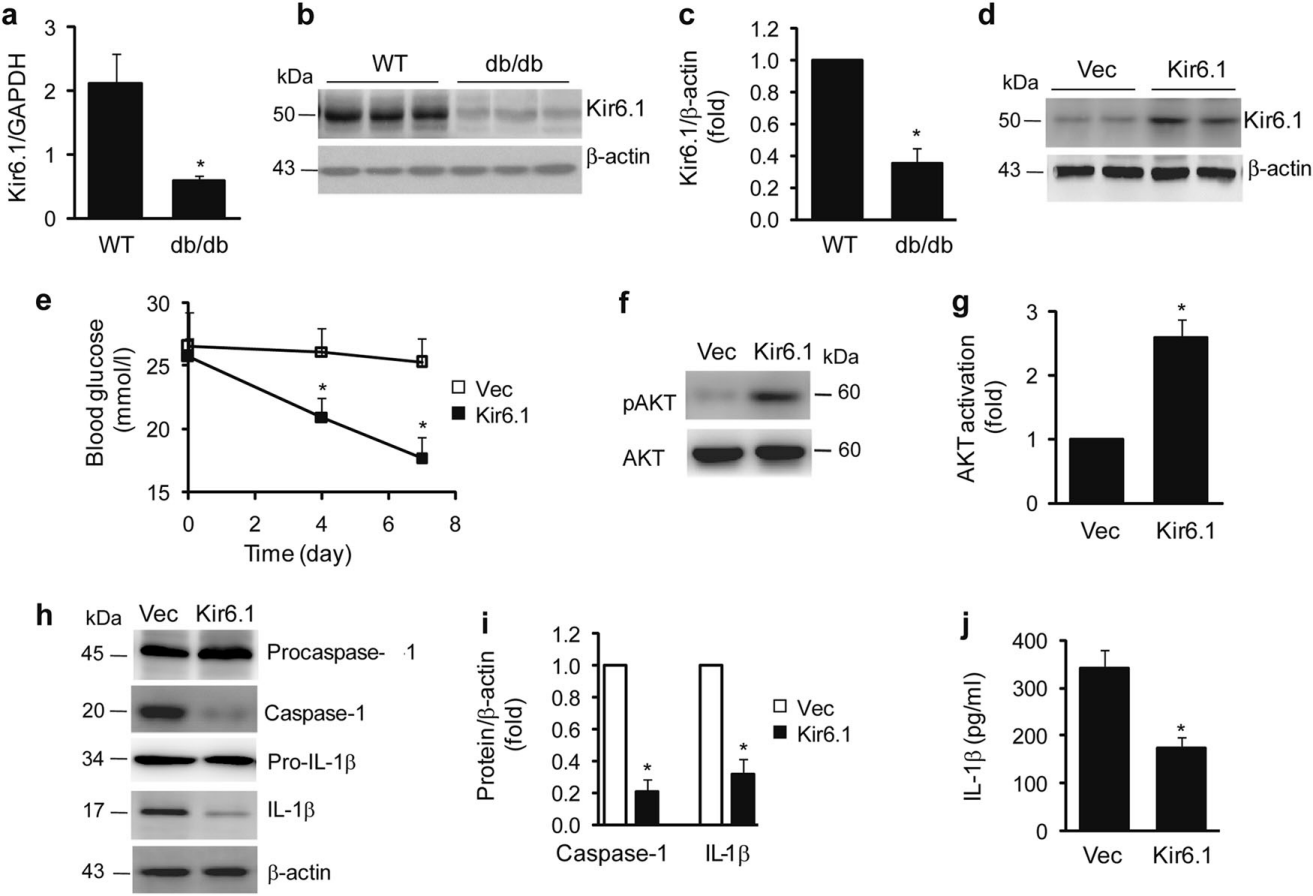
Kir6.1 is downregulated, and rescue of Kir6.1 expression improves insulin resistance in db/db mice. **a** Downregulation of Kir6.1 in the livers of db/db mice detected by qPCR (*n* = 6). **b** Representative immunoblots showing the reduction in Kir6.1 in the livers of db/db mice. **c** Quantification of data shown in (**b**); *n* = 6. The data shown are the mean ± SEM. *p < 0.05 vs WT. **d** Expression of Kir6.1 in the livers of db/db mice after injection with lentivirus for 7 days. **e** Kir6.1 expression reduced the basal blood glucose level in db/db mice; *n* = 6. **f** Representative immunoblots showing that Kir6.1 enhanced the activation of AKT in the liver of db/db mice. **g** Quantification of data shown in (**f**); *n* = 4. **h** Kir6.1 inhibited NLRP3 inflammasome activation in the livers of db/db mice. **i** Quantification of data shown in (**h**). **j** Kir6.1 attenuated serum IL-1β in db/db mice; *n* = 6. The data shown are the mean ± SEM. *p < 0.05 vs vector

Our preceding data demonstrate that Kir6.1 ablation causes activation of the NLRP3 inflammasome and insulin resistance, which can be overcome by transient expression of Kir6.1. We next sought to explore the possible function of Kir6.1 in regulating insulin resistance in db/db mice, a model of spontaneous T2D. We first measured the expression level of Kir6.1 in the liver of db/db mice. The expression of Kir6.1, both at the mRNA and protein level, was reduced by more than 65% in the liver of db/db mice compared to control mice (Fig. 6a–c). Consistent with the data obtained in db/db mice, the expression of Kir6.1 was also decreased in the liver of ob/ob mice and in BMDMs treated with glucose (Fig. s5).

We then determined if the enhanced expression of Kir6.1 could improve insulin sensitivity in db/db mice. Similar to its effect in Kir6.1 KO mice, lentiviral expression of Kir6.1 (Fig. 6d) markedly attenuated blood glucose levels (Fig. 6e), enhanced the activation of AKT in response to insulin stimulation in the liver (Fig. 6f, g), inhibited the activation of caspase-1 in the liver (Fig. 6h, i), and reduced serum IL-1β levels (Fig. 6j) in db/db mice. These results demonstrate that the enhanced expression of Kir6.1 inhibits the NLRP3 inflammasome and improves insulin sensitivity in db/db mice.

### Kir6.1 interacts with NLRP3, and its depletion enhances NLRP3 inflammasome assembly

**Fig. 7:**
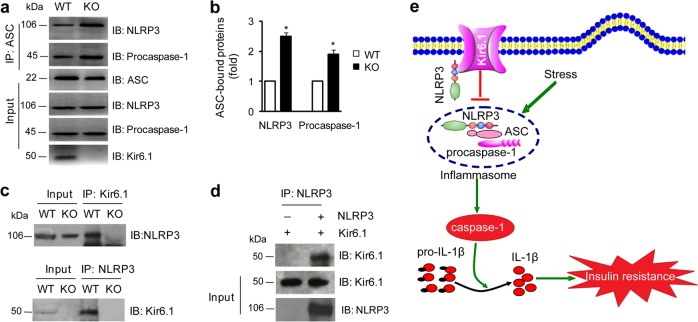
Kir6.1 inhibits NLRP3 inflammasome assembly and interacts with NLRP3. **a** The interaction of ASC with NLRP3 and procaspase-1 in BMDMs isolated from WT and Kir6.1 KO mice assessed by co-IP. **b** Quantification of data shown in (**a**); *n* = 4. The data shown are the mean ± SEM. *p < 0.05 vs WT. **c** The interaction of Kir6.1 with NLRP3 assessed by co-IP in BMDMs isolated from WT and Kir6.1 KO mice. **d** The interaction of Kir6.1 with NLRP3 assessed by co-IP in HEK293T cells transfected with NLRP3 and Kir6.1. **e** A model depicting the roles of Kir6.1, via its interaction with NLRP3, in inhibiting the assembly and activation of the NLRP3 inflammasome, as well as insulin resistance

The assembly of the NLRP3 inflammasome is a prerequisite for its activation^28^. To dissect the molecular mechanisms underlying the function of Kir6.1 in the activation of the NLRP3 inflammasome, we first tested whether Kir6.1 is able to regulate the assembly of the NLRP3 inflammasome by measuring the interaction between the three subunits NLRP3, ASC, and procaspase-1 in co-IP assays. Both NLRP3 and procaspase-1 were clearly detected by immunoblotting in the immunoprecipitates of ASC antibodies in Kir6.1 KO and WT BMBDs (Fig. 7a). However, the amounts of NLRP3 and procaspase-1 in the immunoprecipitates were significantly higher in ATP-stimulated BMBDs from Kir6.1 KO than in BMBDs from WT mice (Fig. 7a, b). These data suggest that the absence of Kir6.1 may enhance the assembly of the NLRP3, ASC, and procaspase-1 subunits into the inflammasome.

We next tested whether Kir6.1 is able to physically associate with the NLRP3 subunit in co-IP assays. Endogenous Kir6.1 was successfully precipitated by NLRP3 antibodies, and endogenous NLRP3 was precipitated by Kir6.1 antibodies in BMBDs from WT but not Kir6.1 KO mice (Fig. 7c). Similarly, Kir6.1 was pulled down by NLRP3 antibodies in human embryonic kidney (HEK) 293T cells, which do not endogenously express the NLRP3 inflammasome subunits and Kir6.1 (Fig. s2), when Kir6.1 and NLRP3 were transiently expressed (Fig. 7d). These results suggest that Kir6.1 may interact with the NLRP3 subunit in the NLRP3 inflammasome.

## Discussion

The most important and surprising finding presented here is that Kir6.1 is a negative regulator of the NLRP3 inflammasome both in mice in vivo and in cultured cells in vitro. Because several studies have unequivocally demonstrated that K^+^ efflux or lowering intracellular K^+^ concentration is a potent trigger for the activation of the NLRP3 inflammasome, our original prediction was that the depletion of Kir6.1 would reduce the formation of K-ATP channels and as such augment intracellular K^+^ concentration, leading to the inhibition of the NLRP3 inflammasome. However, our results clearly demonstrate that Kir6.1 depletion markedly activates the NLRP3 inflammasome, as evidenced by enhanced caspase-1 activation and IL-1β release in Kir6.1 KO mice, as well as in their livers and cultured BMDMs, whereas others have reported that Kir6.1 KO mice show normal inflammasome activation in macrophages^29^. The difference may be due to the different origin of the Kir6.1 KO mouse or due to tissue specificity. These data indicate that under physiological conditions, the expression level of Kir6.1 is essential to maintain the homeostasis of NLRP3.

There are several interesting points regarding the inhibition of the NLRP3 inflammasome by Kir6.1. First, Kir6.1 KO significantly activates the NLRP3 inflammasome without affecting the overall expression of NLRP3, procaspase-1 and pro-IL-1β, suggesting that Kir6.1 is unlikely to regulate the activation of the NLRP3 inflammasome at the transcriptional level or through priming it for activation.

Second, the function of Kir6.1 in inhibiting NLRP3 inflammasome activation is likely specific. The first evidence to support this hypothesis is that Kir6.1 deletion selectively activates the NLRP3 inflammasome but not other inflammasomes, including NLRP1, NLRC4, and AIM2. Another piece of evidence substantiating this notion is that enhanced expression of Kir6.1 inhibits the activation of the NLRP3 inflammasome not only in Kir6.1 KO mice but also in the spontaneous type 2 diabetic db/db mouse model in which Kir6.1 expression is downregulated, and the NLRP3 inflammasome is highly activated.

Third, we demonstrate that transient expression of Kir6.1 is sufficient to inhibit the NLRP3 inflammasome and that Kir6.1 interacts with NLRP3 in cell systems (A549 and HEK293T cells) that do not express endogenous K-ATP channel subunits; thus, Kir6.1 is presumably unable to form the channels. These data suggest that Kir6.1 functions as an integral membrane protein regulating NLRP3 inflammasome activity, rather than via K-ATP channels, to regulate cytosolic K^+^ concentration. These data are also consistent with many reports showing that intracellular K^+^ concentration is mainly under the control of other types of K^+^ channels, such as calcium-regulated BK channels^30,31^.

Fourth, we show that Kir6.1 associates with NLRP3 and Kir6.1 ablation enhances the interaction of the NLRP3 inflammasome subunits as measured in co-IP assays, providing a possible molecular mechanism underlying the function of Kir6.1 in regulating NLRP3 inflammasome function. In addition, we found that the interaction between Kir6.1 and NLRP3 was reduced in response to NLRP3 agonists such as ATP or under the conditions of diabetes. Since a minimal level of NLRP3 expression is required for inflammasome assembly and the expression level of NLRP3 is considered a rate-limiting factor for inflammasome activation^32,33^, the simplest explanation is that the interaction of Kir6.1 with NLRP3 reduces the concentration of free NLRP3, thus inhibiting the assembly of the NLRP3 inflammasome (Fig. 7e). This possibility is also supported by the fact that guanylate-binding protein 5 (GBP5) activates the NLRP3 inflammasome by promoting its assembly^34^. Because Kir6.1 is expressed both at the plasma membrane and in intracellular organelles, such as the ER^35^ and mitochondria^36^, and because ER stress^37,38^ and mitochondrial dysfunction^11,39,40^ are well-described cellular events that activate the NLRP3 inflammasome, we cannot exclude the possibility that Kir6.1 deficiency-induced NLRP3 inflammasome activation is indirectly caused by disruption of the normal function and integrity of intracellular organelles, particularly the ER and mitochondrion.

Fifth, multiple proteins have been demonstrated to be able to regulate NLRP3 inflammasome activation, including double-stranded RNA-dependent protein kinase (PKR)^41^, GBP5^34^ and the NIMA-related kinase Nek7^42^ as positive regulators, and lipid-2^43^, leucine-rich repeat Fli-I-interacting protein 2 (LRRFIP2)^44^, A20^45^, aryl hydrocarbon receptor^32^, Src homology 2 domain-containing tyrosine phosphatase-1 (SHP2)^46^, and small heterodimer partner^47^ as negative regulators. It is interesting to note that Nek7 and PKR interact with NLRP3 to activate the NLRP3 inflammasome. Therefore, the interactions of different regulatory proteins with the components of the NLRP3 inflammasome may produce different effects on NLRP3 inflammasome activation. It should be pointed out that many questions remain unanswered regarding the interaction of Kir6.1 with NLRP3 and the exact role of the interaction in regulating the assembly of the NLRP3 inflammasome. For example, is the interaction between Kir6.1 and NLRP3 direct or indirect? Does the interaction of Kir6.1 and NLRP3 inhibit the interaction of NLRP3 with other inflammasome subunits? Nevertheless, these data suggest that by virtue of its ability to associate with the NLRP3 subunit, Kir6.1 interferes with the assembly and subsequent activation of the NLRP3 inflammasome (Fig. 7e).

Another important finding presented in this paper is the strong evidence indicating that Kir6.1 plays an important role in the pathogenesis of insulin resistance. The direct evidence to support this is the insulin-resistance phenotypes observed in Kir6.1 KO mice, including increased blood glucose and insulin levels, decreased insulin sensitivity and defective glucose metabolism, which can be ameliorated by the rescue of Kir6.1 protein levels. The role of Kir6.1 in insulin resistance is also supported by the fact that increased Kir6.1 expression improves insulin resistance in db/db mice, in which the expression of Kir6.1 is downregulated. These data indicate that the normal expression level of Kir6.1 is essential for the body to respond to the hormone insulin.

It has been demonstrated that excessive activation of the NLRP3 inflammasome impairs glucose metabolism and enhances insulin resistance, which is associated with the onset and progression of metabolic diseases such as T2D^48^. In this study, we demonstrate that Kir6.1 affects the NLRP3 inflammasome and insulin resistance in parallel and, importantly, insulin resistance in Kir6.1 KO mice can be improved by pharmacological inhibition of the NLRP3 inflammasome signaling pathway. Therefore, it is reasonable to conclude that insulin resistance observed in Kir6.1 mice is a consequence of the overactivation of the NLRP3 inflammasome induced by Kir6.1 deletion (Fig. 7e). These results also suggest that enhanced NLRP3 inflammasome activation, which may be caused by Kir6.1 downregulation, is partially responsible for the development of insulin resistance in db/db mice, a genetically manipulated type 2 diabetic mouse model.

Despite extensive investigation, the molecular mechanisms underlying the regulation of activation/inactivation of the NLRP3 inflammasome remain poorly understood. Our studies demonstrate that the K-ATP channel pore-forming subunit Kir6.1 is a bona fide negative regulator of the NLRP3 inflammasome, providing novel insights into the regulatory mechanism of NLRP3 inflammasome activation. These data, together with those of many other studies that have identified a number of positive and negative regulators as discussed above, indicate that the activation/inactivation of the NLRP3 inflammasome is a highly regulated, dynamic process involving many regulatory proteins. The dysfunction of these regulatory proteins may contribute to distinct metabolic diseases, with Kir6.1, as demonstrated here, being an example that leads to insulin resistance and possibly T2D.

In conclusion, our study reveals a previously unrecognized function for Kir6.1 as a negative regulator of the NLRP3 inflammasome and insulin resistance. This function of Kir6.1 is likely mediated through its interaction with NLRP3 to inhibit inflammasome assembly. This study also suggests that Kir6.1 is a promising target for the treatment of inflammasome-mediated inflammatory diseases, such as T2D.

## Supplementary information

Supplementary Materials
