# Supplementary material for: The pore-forming subunit Kir6.1 of the K-ATP channel negatively regulates the NLRP3 inflammasome to control insulin resistance by interacting with NLRP3
Source: Exp Mol Med. 2019 Aug 6;51(8):1–13. doi: 10.1038/s12276-019-0291-6 (PMC6802643; doi:10.1038/s12276-019-0291-6)
Supplement: Supplementary file 1 — Supplementary Materials [file 12276_2019_291_MOESM1_ESM.docx]

**Kir6.1 knockout enhances the PA-induced NLRP3 inflammasome activation in BMDMs.**

**
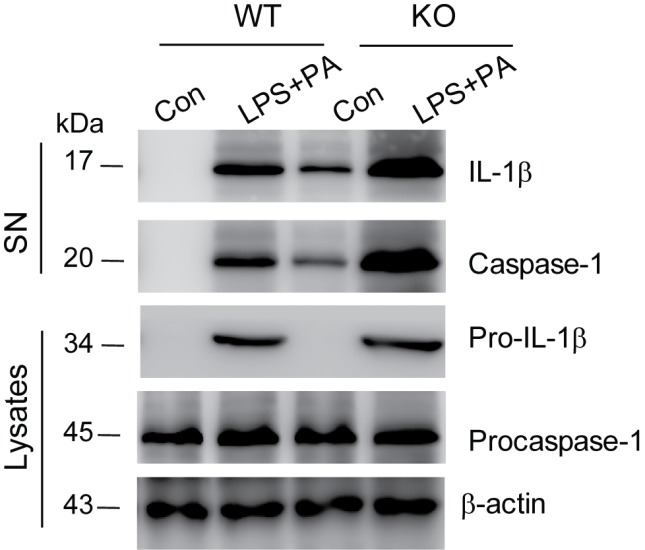
**

**Fig. s1 Kir6.1 knockout enhances the PA-induced NLRP3 inflammasome activation in BMDMs.** Representative immunoblots showing NLRP3 inflammasome activation in response to stimulation with LPS plus palmitic-BSA (PA, 400 μM) for 24 h in MBDMs isolated from WT and Kir6.1 KO mice. The expression of caspase-1 and IL-1β in the cell culture supernatants (SN) and procaspase-1 and pro-IL-1β in the total cell lysates were measured by immunoblotting. n=3.

**Kir6.1 deficiency does not affect the activation of the NLRP1, AIM2 and NLRC4 inflammasomes in BMDMs**

**
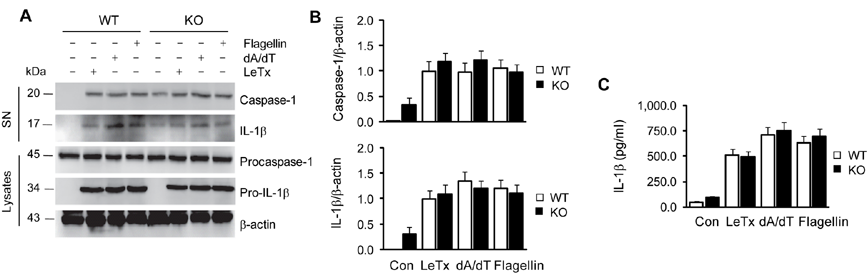
**

**Fig. s2 Kir6.1 deficiency does not affect the activation of the NLRP1, AIM2 and NLRC4 inflammasomes in BMDMs. (A)** BMDMs isolated from WT and Kir6.1 KO mice were cultured and then stimulated with flagellin, dA/dT or LeTx. The expression of caspase-1 and IL-1β in the cell culture supernatants (SN) and procaspase-1 and pro-IL-1β in the total cell lysates were measured by immunoblotting. **(B)** Quantification of caspase-1 (upper panel) and IL-1β (lower panel) as shown in (**A**). **(C)** IL-1β expression in the supernatants measured by ELISA. The data shown are the mean ± SEM from four independent experiments.

**A549 and HEK293 cells do not express endogenous K-ATP channel subunits**


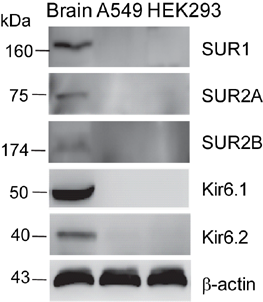


**Fig. s3. A549 and HEK293 cells do not express endogenous K-ATP channel subunits.** A549 and KEK293 were cultured and the total cell lysates prepared. The expression of SUR1, SUR2A, SUR2B, Kir6.1 and Kir6.2 was measured by immunoblotting. A sample prepared from the mouse brain was used as a positive control. n=3.

**Kir6.1 knockout does not affect the intracellular K^+^ level in BMDMs**


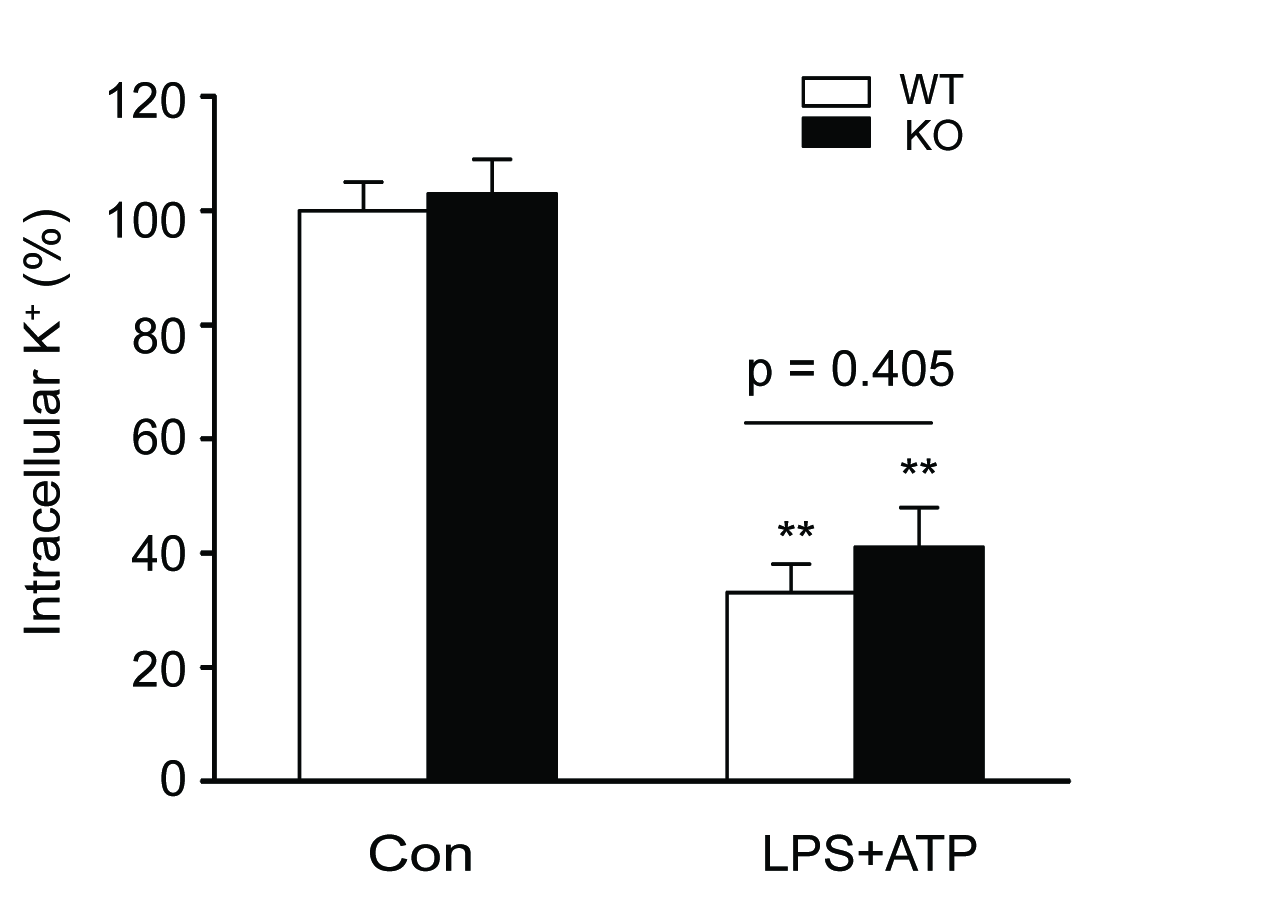


**Fig. s4. The intracellular content of K^+^ in WT and Kir6.1 KO BMDMs after treatment with LPS + ATP was measured by ICP-OES.** The data shown are the mean ± SEM from three independent experiments. ^**^p<0.01 vs corresponding control group.

**The expression of Kir6.1 is reduced in liver of ob/ob mice and in BMDMs treated with glucose.**


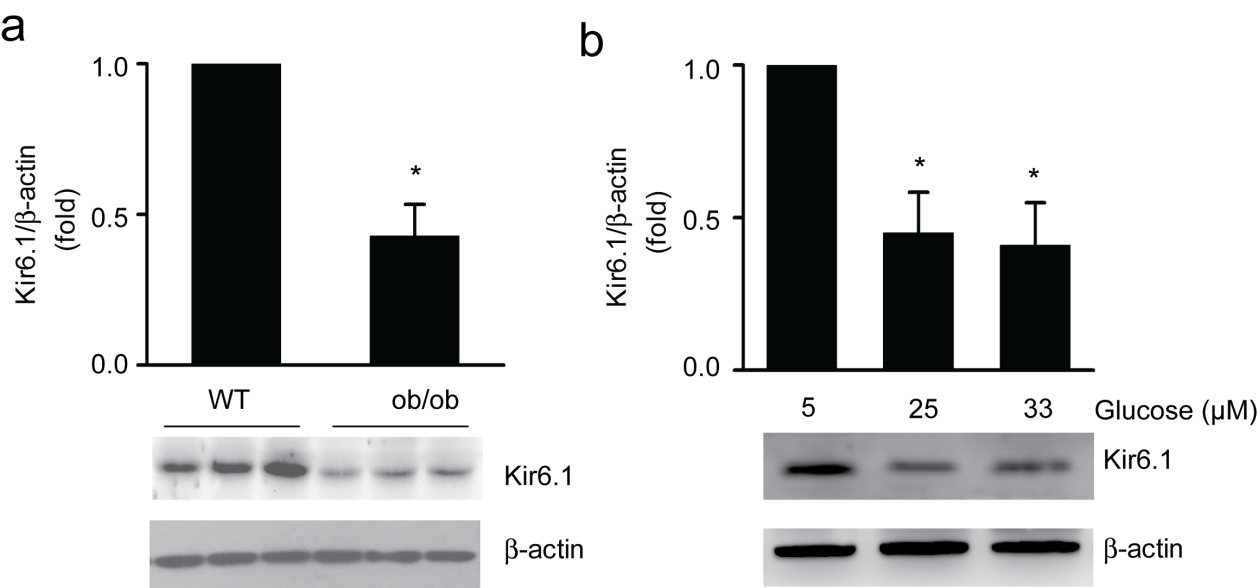


**Fig. s5. The expression of Kir6.1 is reduced in liver of ob/ob mice and in BMDMs treated with glucose.** **a-b,** Representative immunoblots and quantification of Kir6.1 expression in liver of ob/ob mice (n=6, **a**) and in BMDMs treated with different concentrations of glucose (**b**). The data shown are the mean ± SEM from three independent experiments. ^*^p<0.05 vs WT mice; ^*^p<0.05 vs 5 μM glucose group.
